# Effect of Aging and Retail Display Conditions on the Color and Oxidant/Antioxidant Status of Beef from Steers Finished with DG-Supplemented Diets

**DOI:** 10.3390/foods11060884

**Published:** 2022-03-20

**Authors:** Manuela Merayo, Sergio Aníbal Rizzo, Luciana Rossetti, Dario Pighin, Gabriela Grigioni

**Affiliations:** 1 Instituto Tecnología de Alimentos—Instituto de Ciencia y Tecnología de Sistemas Alimentarios Sustentables, UEDD Instituto Nacional de Tecnología Agropecuaria—Consejo Nacional de Investigaciones Científicas y Técnicas, CC 25, Castelar 1712, Argentina; merayo.manuela@inta.gob.ar (M.M.); rizzo.sergio@inta.gob.ar (S.A.R.); rossetti.luciana@inta.gob.ar (L.R.); grigioni.gabriela@inta.gob.ar (G.G.); 2 Facultad de Ciencias Médicas, Pontificia Universidad Católica Argentina, Alicia M. de Justo 1500, Ciudad Autónoma de Buenos Aires 1425, Argentina; 3 Escuela Superior de Ingeniería, Informática y Ciencias Agroalimentarias, Universidad de Morón, Cabildo 134, Morón 1708, Argentina

**Keywords:** beef, distiller grains, antioxidants, oxidative stability, color

## Abstract

The aim of this work was to study the effect of finishing diets including distiller grains (DG) on color and oxidative stability of beef after being exposed to aerobic retail display conditions, with or without previous aging. For this purpose, beef samples from animals fed with finishing diets including 0%, 15%, 30%, and 45% DG (on a dry matter basis), which had been exposed to aerobic retail display conditions, with or without previous aging under vacuum packaging, were evaluated. The content of γ-tocopherol, β-carotene, and lutein in diet samples increased with the level of DG. Beef evaluated at 72 h post-mortem showed greater content of γ-tocopherol and retinol as the DG level increased. Meat color was not affected by DG inclusion, but color parameters decreased with retail conditions. Meat from animals fed with DG showed the lowest values of thiobarbituric acid reactive substances (TBARS), independently of the retail display conditions. However, all samples were below the threshold associated with rancid aromas and above the a* value related to meat color acceptance. Thus, feeding diets including up to 45% of DG improved the antioxidant status of meat, preserving the color, and delaying lipid oxidation in meat samples under the display conditions evaluated.

## 1. Introduction

The bioethanol industry has been an important contributor to country economies since it has supplied renewable energy sources and has demanded biobased feedstocks [1]. Some countries, like the USA, Brazil, and Canada, have a long history in bioethanol production and they shared, in 2021, 84% of world production [2], while other countries, like Argentina, have produced bioethanol more recently, using corn as a feedstock. Distiller grains (DG), which are the byproduct of the bioethanol industry, are considered a valuable supplement in animal feeding because they are rich in the fat, protein, and fiber portion of corn grain [3,4]. Several works have analyzed the effect of feeding with DG on beef pro-oxidative components, like unsaturated fatty acids and lipid oxidation [3]. However, few reports have focused on the effect of DG on beef anti and pro-oxidant balance and its relationship with beef color and lipid oxidation. During meat storage, lipid oxidation could be influenced by the packaging and ambient conditions, and intrinsic meat characteristics, like the balance of anti- and pro-oxidants content and the abundance of unsaturated fatty acids [5]. This process, together with microbial spoilage, is the main cause of meat quality deterioration, affecting color, flavor, and nutritional value [6,7]. Previous works have stated that there is a relationship between meat color deterioration and lipid oxidation since the biochemical reactions responsible for both myoglobin and lipid oxidation generate products that can accelerate oxidation processes reciprocally [5]. Moreover, it is known that meat visual appearance plays an important role in consumers’ choices [8]. This visual appearance depends on physical and chemical factors, including the meat color, which is one of the most important attributes evaluated by consumers at the purchase point [8,9,10]. Meat color could be influenced by a variety of factors related to animal production and commercialization strategies, including aging and retail display conditions [9,11]. Meat visual appearance can be compromised by discoloration, which is related to oxymyoglobin oxidization to metmyoglobin [5,11].

Oxidative processes could be delayed through different strategies, including the addition of antioxidant compounds into the meat product, adding these compounds directly on the meat surface or through active packaging, and including antioxidant compounds in the feeding diets of the animals [6,12,13]. Among antioxidant compounds, α-tocopherol has been demonstrated to be effective in preserving color in beef, while it has an important effect on lipid oxidation delay [9]. In addition, dietary delivery of α-tocopherol has been shown to be more effective than its exogenous addition due to more efficient antioxidant incorporation through cell membranes [5,13,14]. Among feeding diets, there are plenty of strategies targeted either to improve meat quality, like supplementation with antioxidant compounds or the inclusion of feeding sources of n-3 polyunsaturated fatty acids (PUFAs) or to reduce the cost of the diet, like the inclusion of agro-industrial byproducts [7,15]. In this sense, several studies have demonstrated that DG supplementation increased PUFA content of beef and lipid oxidation during retail display conditions [16,17], while other studies have found no effect of DG inclusion on lipid oxidation [18,19]. These divergencies could be explained by the complex balance required between antioxidant and pro-oxidant compounds present in meat. Therefore, the aim of the present study was to evaluate the effect of finishing diets containing increasing levels (15, 30, and 45%) of wet DG with solubles on color and oxidative stability of *Longissimus thoracis et lumborum* (LTL) steaks after being exposed to aerobic retail display conditions, with or without previous aging under vacuum packaging.

Our findings indicate that beef from DG diets had greater antioxidant status than beef from diets without DG inclusion, which positively impacts the balance between anti- and pro-oxidant compounds and meat oxidative stability during simulated commercial conditions.

## 2. Materials and Methods

### 2.1. Experimental Design and Diets

Meat samples (LTL) were obtained from yearling Angus steers that had been fed with finishing diets including increasing levels of wet DG with solubles. For this purpose, a total of thirty-six weaned steers (initial live weight, 191 ± 12 kg) were randomly allocated to 12 pens (three steers per pen) and fed with a high-concentrate diet for three weeks to adapt to the feeding system. Then, the pens (3 pens per treatment) were randomly assigned to one of the four dietary treatments. Steers were fed for 70 days. The control diet consisted of cracked corn grain, soybean meal, and alfalfa hay. The dietary treatments consisted of four levels of inclusion of DG, replacing the corn and soybean meal: 0% (0DG), 15% (15DG), 30% (30DG), or 45% (45DG) (dry matter (DM) basis; Table 1). The diets were formulated to be isoenergetic (2.95 Mcal/kg DM) and isoproteic (14.9%), except for 45DG which supplied 17.9%. Three samples of each diet were collected during the feeding, vacuum packed, and stored in darkness until analysis. No effect of feeding diets on animal performance during the trial was observed and animals showed an average daily gain of 1.586 ± 0.045 kg/d. Details of the feeding diets and cattle performance have been previously reported [20,21].

When animals reached slaughter conditions (commercial endpoint based on visual appraisal and final body weight of 316 ± 14 kg on average), they were transported to a licensed commercial abattoir in a single group. Animals were slaughtered according to standard commercial procedures on the same day after mixing groups to avoid peri-slaughter effects. Carcasses were suspended through the Achilles tendon and were not electrically stimulated.

### 2.2. Sample Preparation

After slaughter, carcasses were individually graded and chilled at 4 °C. Forty-eight hours later, a section of the LTL from the 13th rib region was removed from the striploin of each carcass and transported under refrigeration to the Food Technology Institute of the National Institute of Agricultural Technology (INTA), Buenos Aires, Argentina. At 72 h post-mortem, two steaks (2.5 cm thick each) from each rib section were stored at one of the following retail display conditions that resembled commercial local practice:

- R1: the steaks were individually placed on Styrofoam trays, overwrapped with oxygen-permeable polyvinylchloride film, and placed under refrigeration at 4 ± 2 °C for four days in darkness and then for three days with 7 h of illumination (D65, 700 lux) per day.
- R2: the steaks were placed under vacuum-packed conditions at 1 ± 1 °C and darkness for 25 days, plus a retail display period under aerobic exposure. For this last stage, the steaks were placed on a Styrofoam tray overwrapped with oxygen-permeable polyvinylchloride film for three days under refrigeration at 4 ± 2 °C and with 7 h of illumination (D65, 700 lux) per day.

Other steaks from each rib section were stored at −80 °C until further analysis.

### 2.3. α- and γ-Tocopherol, β-Carotene, Retinol and Lutein Content

The contents of α-tocopherol, γ-tocopherol, β-carotene, α-carotene, retinol, and lutein from feedstuffs and meat samples were determined as described by Buttriss and Diplock [22] with modifications by Descalzo et al. [23]. Briefly, 5 g of lean tissue was placed in a plastic conical tube containing 10 mL of phosphate buffer (0.05 M; pH 7.7) and homogenized for 2 min at 3000 rpm with an Ultraturrax T25 homogenizer (IKA, Darmstadt, Germany). Aliquots of 1 g of homogenate were placed into a screwcap test tube with 3 mL of pyrogallol 1% in ethanol. Thereafter, 0.3 mL of KOH 12 N in water was added to each tube for saponification. The tube contents were mixed by vortexing for 30 s, and placed in a stirred water bath for 30 min at 70 °C. After cooling, 1 mL of water was added to each tube. Following the addition of 5 mL of n-hexane, the samples were mixed by vortexing for 2 min; the upper hexane layer was then transferred into a new screw cap tube and the aqueous phase was reextracted with 5 mL of n-hexane. The combined extracts were taken to dryness under a dry nitrogen gas stream, and the residue was dissolved in 500 μL of absolute ethanol (J.T. Baker, Mexico, HPLC grade) and filtered through a 0.45 μm-pore nylon membrane before injection of samples. All samples and standards (external standards for each vitamin) were analyzed by reverse-phase high-performance liquid chromatography (HPLC) using a Thermo Scientific Dionex UltiMate 3000 RS system consisting of a quaternary pump with a membrane vacuum degasser connected to an auto-sampler with an injection loop (10 to 100 μL) and a C18 column (250 × 4.6 mm i.d., Alltima, 5 μm particle size; Alltech, Argentina) fitted with a guard column (Security GuardAlltima C18, Alltech, Argentina). The mobile phase was ethanol:methanol (60:40, *v*/*v*) at a flow rate of 1 mL/min. The technique was optimized to determine tocopherols, carotenoids, and retinol within the same elution time of 25 min. Tocopherols were detected by fluorescence at λ_ex_ = 296 and λ_em_ = 330 nm. A diode array detector was set at λ = 445 nm and λ = 325 nm for the detection of carotenoids and retinol, respectively. Calibration curves were performed with DL-α-tocopherol (Merck, Darmstadt, Germany), γ-tocopherol, β-carotene, lutein, and retinol standards (Sigma-Aldrich, St. Louis, MO, USA) diluted in ethanol. Chromatograms were recorded using the Chromeleon 6 software.

For feedstuff samples, the methodology was identical, except that the samples were diluted 1 to 10 with ethanol before injection.

### 2.4. Muscle Ferric Reducing Antioxidant Power (FRAP)

The FRAP assay applied to meat samples measures endogenous ions that could react with tripyridyltriazine (TPTZ) and develop blue color. Following the procedure described by Ou et al. [24] and modified by Descalzo et al. [25], 5-g chopped meat samples were disrupted for 2 min at 3000 rpm with an Ultraturrax homogenizer (IKA, Staufen, Germany) in 10 mL of potassium phosphate buffer (0.05 M, pH 7.7). Homogenates were centrifuged at 10,000× *g* for 30 min at 4 °C and the supernatant was collected. Then, 83-µL aliquots of supernatant were added to 2.5 mL of FRAP buffer containing 10 mM TPTZ, 40 mM HCl and 20 mM Fe_2_Cl_3_ (Sigma-Aldrich, St. Louis, MO, USA) added to 300 mM acetate buffer. The reaction mixture was incubated for 5 min at 37 °C in a water bath and then cooled in an ice-water bath for 10 min. Immediately after, samples were measured at λ = 593 nm (Spectrometer UV–vis-BIO Lambda 20, Perkin Elmer). Another 83-µL aliquots of supernatant were added to 2.5 mL of TPTZ/HCl solution, without the addition of Fe_2_Cl_3_, to determine endogenous Fe^+2^ content. The FRAP activity of the samples was measured against a calibration curve made with ferrous sulfate (Fe_2_SO_4_·7H_2_O, Sigma-Aldrich de Argentina SA) within a range from 100 to 1000 µM, and results were expressed as Fe^+2^ equivalent in µM.

### 2.5. Thiobarbituric Acid Reactive Substances (TBARS)

TBARS were analyzed by the steam distillation method, as described by Pensel [26] with modifications of Descalzo et al. [23], and expressed as mg of malonaldehyde (MDA) per kg of lean muscle. Briefly, triplicate aliquots (5 g) of meat were chopped and processed in a stomacher-type homogenizer for 2 min in bags containing 12.5 mL of trichloroacetic acid (Merck, Darmstadt, Germany) solution (20% *w*/*v*) in 1.6% metaphosphoric acid. Then, 12.5 mL of water was added, and the mixture was processed for another 30 s. Slurries were filtered and aliquots of 5 mL were separated. An equal volume (5 mL) of 0.02 M 2-thiobarbituric acid (Sigma-Aldrich, St. Louis, MO, USA) was added. Samples were incubated at 80 °C for 1 h until a pink color was developed. Color intensity was determined at maximum absorption, λ = 530 nm, and TBARS concentrations were calculated from a calibration curve by using 1,1,3,3-tetraethoxypropane (Sigma-Aldrich, St. Louis, MO, USA) as standard within a range from 0 to 0.5 µM.

### 2.6. Meat Color

Meat color was assessed using the CIELab system, which provides the color parameters L* (lightness, from black to white), a* (redness, from green to red), and b* (yellowness, from blue to yellow). The chroma parameter was calculated according to the American Meat Science Association (AMSA) [27]. Measurements were carried out with a Minolta CR-400 colorimeter (Konica Minolta Sensing, Inc., Bergen, NJ, USA) as described by the AMSA [27]. The instrumental conditions used were artificial D65 illuminant, 8 mm port size, and a two-degree standard angle observer. The instrument was calibrated against a white plate (Y = 93.8, x = 0.3155, y = 0.3319). Each sample was allowed to bloom for 45 min at 4 °C prior to the first measurement, and six scans of each steak were averaged for statistical analysis.

### 2.7. Statistical Analysis

Statistical analysis was conducted using InfoStat Software version 2018e [28]. Data normality was checked with the Shapiro-Wilk test and homogeneity of variances with the Levene test. Data that did not show a normal distribution or homogeneity of variances (*p*-value < 0.05) were analyzed with the Kruskal-Wallis test. Data from diet composition were analyzed as a completely randomized design with the level of DG inclusion in the diets as the main effect. Data from meat quality at 72 h post-mortem were analyzed as a split-plot design where pens were the whole plot and the level of DG in the diets was the split-plot. Linear and quadratic relationships were detected by response curves. Data from the retail display were analyzed as a split-split plot design where pens were the whole plot, the level of DG in the diets was the split-plot and the conditions of the retail display were the split-split plot. The least significant differences were set at a 5% level and means were compared by Tukey’s test. Principal component analysis (PCA) was applied to the data of beef parameters in each retail display condition, using SPSS 13.0, following [29].

## 3. Results and Discussion

### 3.1. Content and Animal Intake of Antioxidant Compounds in Feedstuffs

Corn is a good source of fat-soluble antioxidant compounds, such as vitamin E and carotenoids [30,31]. Carotenoids are composed of carotenes and xanthophylls, which provide yellow color to animal products [30,31]. The co-products obtained after corn processing, like DG, increase the concentration of corn nutrients, except for sugars [3]. Previous reports have indicated that samples of dried DG with solubles obtained from different plants of bioethanol production have, on average, two-fold higher content of α-tocopherol, γ-tocopherol, and lutein than corn grain [30].

In our study, the content of α-tocopherol was almost three times greater in the dietary treatments with DG inclusion (Table 2) than in the control diet. Other authors have reported that the content of α-tocopherol was 8.0 and 7.4 µg/g in corn-based diets supplemented with 20 and 40% DG respectively and no differences were seen when compared with dry-rolled corn control diet (9.3 µg/g) [32].

Regarding γ-tocopherol, β-carotene, and lutein, their contents increased with the level of DG inclusion in the diets (Table 2). To our knowledge, previous works had analyzed only the content of these compounds in DG alone, as an ingredient, but not in feedstuffs that included DG. Therefore, the values obtained in this work were compared with values reported for complete diets based on corn grain. The values selected for the comparison were those reported by Blanco et al. [33] for total mixed ration (TMR) and corn diets and those by Pouzo et al. [34] for corn diets. The values of lutein observed were similar to those reported for TMR (10.1 µg/g DM), whereas those of γ-tocopherol observed in 15DG, 30DG, and 45DG were higher than the values reported for the corn diet (79.5 µg/g DM, [33] and 14.1 µg/g DM, [34]). Regarding β-carotene, the values observed in all dietary treatments in this study were greater than those reported for the corn diet (1.47 µg/g DM, [34]).

**Figure 1:**
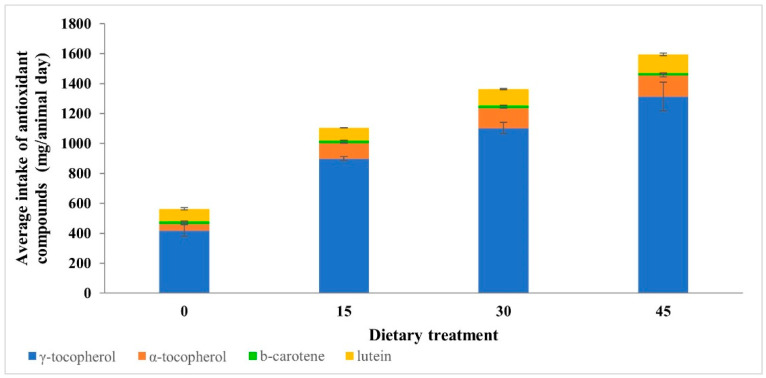
Average intake (mean ± SD) of antioxidant compounds weighted with the consumption of the animals, for each dietary treatment (0, 0% DG control; 15, 15% DG; 30, 30%DG; 45, 45% DG, DM basis).

To estimate the vitamin intake of the animals, the composition of the feeding diets was weighted with the consumption of animals (Figure 1). The DM consumption of repetitions and treatments was measured every other week [20] and an average of consumption was calculated for each diet: 8.64 DM/day for 0DG, 8.77 DM/day for 15DG, 8.31 DM/day for 30DG, and 8.17 DM/day for 45DG. No statistical analysis was done on these data since DM intake was recorded on a group basis. Results showed that the intake of antioxidant compounds, except for that of β-carotene, numerically increased with the level of DG inclusion in the diets.

### 3.2. Antioxidant Compounds in Fresh LTL Muscle

The diet composition influences animal metabolism, use, and storage of glycogen and accumulation of anti- and pro-oxidant compounds [13,35]. Thus, the feeding diets could affect the content of antioxidant compounds in meat and its susceptibility to oxidative reactions. In our study, the content of γ-tocopherol in meat samples increased with the level of DG (Table 3), which could be explained by the fact that the feeding diets supplied greater content of γ-tocopherol and that the intake of animals increased with the level of DG inclusion. Besides, the content of γ-tocopherol observed was greater than that reported by Pouzo et al. [34], when feeding diets were supplemented with flaxseed.

It has been reported that the supplementation of feeding diets with vitamin E increases the concentration of α-tocopherol in meat [13,32], and that feeding diets including less than 200 µg/g of α-tocopherol produce lamb meat with 1.97 µg/g of α-tocopherol, on average [36]. In our study, although feeding diets were not supplemented with vitamin E, a numerical increase of α-tocopherol was observed in meat samples as the level of DG increased. Salami et al. [37] have recently shown no effect of the inclusion of DG in finishing diets on the content of α-tocopherol in meat. These authors reported that the α-tocopherol content was 2.57 μg/g muscle, on average, i.e., greater than the values observed in our study. However, in their case, the supplementation of DG was in combination with *ad libitum* grass silage, which provides greater amounts of α-tocopherol than grain [13]. Other authors have reported that ground beef from animals fed with 20 and 40% of DG in corn-based diets had 1.70 and 1.79 μg/g of α-tocopherol, respectively [32]. In our work, the content of α-tocopherol was lower than the value reported by Da Silva Hampel et al. [36] in the meat of lambs fed with 0–200 µg/g of tocopherol level in the diet; it was only similar (1.41 μg/g) in beef samples from 45DG dietary treatment, which supplied 17.27 μg/g of α-tocopherol.

In addition to tocopherol, feeding diets supplied carotenoid compounds. These compounds are converted to retinol in the animal and exert antioxidant capacity and contribute to the yellowness of subcutaneous fat [6,12]. The content of carotenoid compounds in meat is highly variable since their incorporation depends on their content in the feeding diets, and the accumulation in adipose tissue, which is related to the type of muscle and the individual uptake capacity of the animal [13,35]. Previously, it has been reported that the content of carotenoids and their retinoid derivatives is at least one order below that of α-tocopherol [13]. In fact, Pouzo et al. [34] reported that meat samples from grazing systems supplemented with corn had 0.09 ug of retinol/g of fresh meat and 1.15 ug of α-tocopherol/g of fresh meat. In our study, the values of retinol observed in meat tended to be greater in samples from DG diets. Additionally, the values observed were one order below the values of α-tocopherol, independently of the dietary treatment (Table 3).

Neither the antioxidant capacity, measured as FRAP, which determines the total reducing capacity of antioxidant compounds nor the lipid oxidation, measured as TBARS, in meat samples at 72 h post-mortem were affected by the level of DG inclusion in the diets (Table 3). In general terms, no differences were seen in meat oxidation stability due to the dietary treatment. Additionally, other authors have observed a lack of effect on lipid oxidation in meat from steers fed 50% DG and aged for 2 days [38]. Interestingly, recently published data have shown that meat from cattle finished on a grass silage-based diet in combination with corn or wheat DG supplementation showed no differences in the FRAP values, after 14 days of aging, regardless of the source of DG [37].

### 3.3. Antioxidant Compounds, Oxidation and Color Stability of LTL under Storage Conditions

The content of antioxidant compounds observed in LTL samples stored at two retail display conditions is shown in Table 4. No significant interactions were observed between the main effects.

The content of α-tocopherol was not affected either by the feeding diet or the storage conditions, and the average concentration was 0.58 µg/g meat. Regarding γ-tocopherol, a higher content was observed in meat from steers fed with DG diets, while it was not affected by the storage conditions. However, in all cases, the values of α- and γ-tocopherol observed were lower than those observed at 72 h post-mortem. Previously, it has been reported that vitamin E affects lipid and color stability after refrigerated storage [13]. In the present study, during storage, α-tocopherol levels dropped around 34% in meat from all diets, except in those from 45DG, in which it decreased by 64%, whereas γ-tocopherol levels dropped around 20% in meat from all diets, except in those from 45DG, in which it decreased by 48%. Indeed, the FRAP assay showed that the total reducing activity in meat was greater for R1 than for R2, but also that it was not affected by the feeding diets. Other authors have reported that, after meat aging and aerobic exposure, both the FRAP values and the content of antioxidant vitamins decreased [34]. In our study, the effect of storage on FRAP was not observed on vitamin content.

Regarding retinol content, the meat from animals fed 30DG had greater content than that from animals fed 15DG and 45DG, while the meat from 0DG had the lowest content (Table 4). Retinol content was not affected by the retail display conditions.

**Figure 2:**
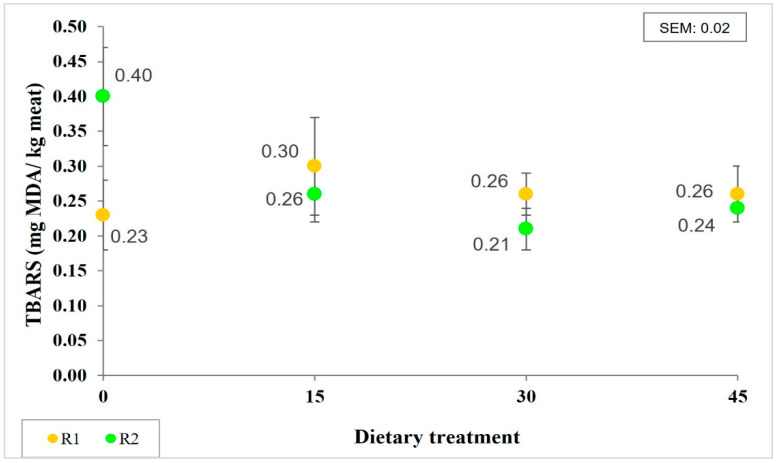
Lipid oxidation (mean ± SEM) measured as thiobarbituric acid reactive substances (TBARS) in LTL at the end of storage and retail display conditions for each dietary treatment (0, 0%DG control; 15, 15% DG; 30, 30%DG; 45, 45% DG, DM basis).

Together with α-tocopherol, other compounds like the minor forms of vitamin E, carotenoids, and their retinoid derivatives, protect tissues against free radicals and prevent oxidation reactions [13]. All these compounds could be administered in the feeding diets, as stated before. However, pro-oxidant compounds, like unsaturated fatty acids, could also be administered in the diets. It has been reported that, during refrigerated storage, oxidative processes increase exponentially [13], since factors like temperature, the presence of light, and oxygen act as catalysts [6,9]. In our study, the values of TBARS measured at the end of the storage period in LTL samples showed a tendency (*p* = 0.08) to interact between dietary treatment and retail display conditions (Figure 2). Meat from DG diets showed similar TBARS values, independently of the storage condition. Interestingly, the meat from 0DG showed the highest TBARS values when exposed to R2 conditions. In this sense, greater TBARS values have been reported in meat samples from 0% DG diets, compared to meat samples from 50% DG diets, after 21 days of aging [38]. However, de Mello et al. [17] observed greater TBARS values in meat from animals fed 30% DG than in meat from animals fed 0 and 15% DG and aged for 42 days. These authors observed meat surface discoloration after aging followed by a retail display and related these events with a higher content of PUFAs in meat when feeding cattle with 30% DG.

The TBARS assay is used as a marker of lipid oxidation and as a predictor of the perception of rancidity in meat. In different studies, values of 2–2.5 mg MDA/kg have been established as the accepted limit at which meat shows no rancidity [6]. In our study, all the TBARS values observed were below that threshold.

As stated above, anti- and pro-oxidants can be provided in the feeding diets, and their balance would be a determinant for the oxidative stability of meat. In our study, the feeding diets supplied liposoluble vitamins to meat, mostly γ-tocopherol. However, α-tocopherol is the isomer with the greatest antioxidant action and at least 3.0 to 3.5 µg/g of meat is needed to preserve tissues from oxidation and/or to maintain color stability [7,23,39]. Interestingly, it has been proposed that a dosage level of γ-tocopherol and a similar dosage level of α-tocopherol are approximately equally effective in preventing oxidation in red meat [39]. In our study, the sum of the content of antioxidant vitamins (1.39 µg/g meat on average) was below the optimal levels proposed to preserve tissues from oxidation, but the extent of oxidative damage was far below the threshold for rancidity. Therefore, it could be assumed that the antioxidant compounds present in meat samples were exerting a cooperative antioxidant activity.

It is known that the nutritional strategies that modify the fatty acid profile of meat could increase the susceptibility of meat to lipid oxidation by increasing n-3 PUFAs [7,13]. We showed that the content of PUFAs and n-6 PUFAs did not change with the DG level, while that of n-3 PUFAs decreased numerically [40]. This effect was statistically seen in the n-6/n-3 PUFA ratio, which increased as the DG level increased due to the decrease in n-3 PUFAs. In the present study, we observed that the inclusion of DG in the feeding diets supplied both more antioxidant compounds and fewer pro-oxidant compounds.

In this sense, it has been reported that the supplementation of DG diets with vitamin E does not improve the effect against lipid oxidation, since the meat from 30DG-diets, with and without vitamin E supplementation, had similar values of TBARS after 7 days of retail display [41].

The meat color parameters found at the end of the retail display conditions are presented in Table 5. In the case of the R2 storage condition, an extra measurement was done at the end of the vacuum-packed storage, prior to display conditions (R2_a_). The color parameters analyzed showed no interaction between the main effects.

Meat color did not differ statistically with the inclusion of increasing levels of DG in the diets. These results are in agreement with those presented by de Mello et al. [17], who compared beef from DG diets with control diets (0% only corn, 15% and 30% DG). On the other hand, Depenbusch et al. [19] reported a linear decrease in redness (a* value), after 7 days of retail display conditions, in steaks from heifers fed increasing levels of DG (0% to 75%). Moreover, to analyze differences in beef color, delta color change (ΔE) was calculated, following AMSA procedures, between steaks from DG diets and steaks from the control diet. The values of ΔE obtained for 15DG, 30DG, and 45 DG were 1.58, 1.69, and 2.13, respectively. Some authors reported that values of ΔE from 2 to 10 indicate that differences in color are perceptible by the human eye at a quick look [42] while other authors reported that values between 1.5 to 3.0 in beef could be evident to consumers [43].

Meat color stability is the result of the balance between anti- and pro-oxidant compounds [44]. It could be assumed that meat with an increase in PUFA content, due to the inclusion of DG in the diet, would be more susceptible to lipid oxidation and discoloration. Thus, as previously reviewed, it is difficult to define a general effect on meat color due to the inclusion of DG in the diet [3].

During storage, the formation of metmyoglobin (MMb) modifies meat color with a decrease in both a* and C* parameters [45]. Besides, the rate at which MMb develops could be affected by the conditions of packaging and storage. In the present study, storage conditions influenced color parameters. Samples from R2 conditions were brighter and had higher values of a* and C* than steaks from R1 conditions. Indeed, samples from R1 and R2 showed a value of ΔE of 2.93, which indicated that differences could be evident to the human eye [42,43]. Additionally, a decrease in a* and C* values was observed in R2_b_ with respect to R2_a_.

Meat from R1 had lower values of color parameter than meat from R2_b_ (Table 5). These results are in accordance with those of Jose and McGilchrist [46], who proposed that in low pH beef, aging increased bloom depth and made meat appear brighter and redder in color. However, other authors reported that aging time before retail display did not affect the redness (a* parameter) of meat samples from DG diets, while retail display conditions decreased the values of a* [41]. Similarly, in our study, a decrease in a* and C* values were observed when changing from vacuum to aerobic packaging in R2 (R2_b_ with respect to R2_a_). This decrease could be associated with an increase in MMb content when samples were exposed to aerobic conditions.

In this sense, it has been reported that during refrigeration storage, lipid oxidation occurs more slowly than discoloration [14]. McKenna et al. [47] reported that color-stable muscles had lower TBARS than less color-stable ones. These authors found values up to 0.35 mg/kg for *M. longissimus lumborum* and *M. longissimus thoracis* after 5 days of a retail display while preserving color traits. In agreement, in our study, the values of TBARS observed were lower than 0.35 mg/kg, except for meat from the 0DG treatment at R2 storage conditions. Furthermore, the values of a* measured for all samples in our study were above 14.5, the value considered as a threshold for beef color acceptability [48].

**Figure 3:**
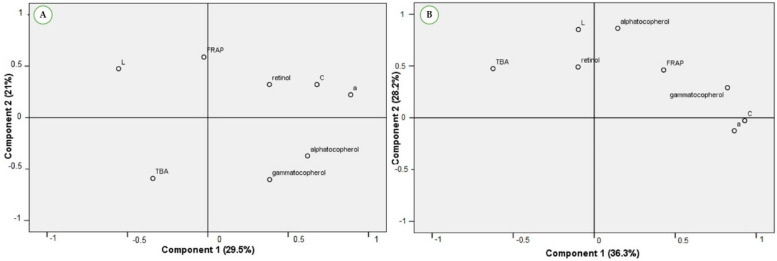
Principal component analysis (PCA) depicting the relationship between the oxidant/antioxidant status and the color parameters, at (**A**) R1 conditions (aerobic exposure for seven days) and at (**B**) R2 conditions (vacuum-packed storage for 25 days, plus aerobic exposure for three days).

Moreover, a PCA was performed to depict the relationship between the oxidant/antioxidant status and the color parameters, at each storage condition (Figure 3). The new components identify where the maximum variance of the data occurs in a multidimensional space. For the R1 condition, the first component (PC1) accounted for 29.5% of the variance, the second component (PC2) accounted for 21.0% of the variance, and the third component (PC3) accounted for 19.3% of the variance. For R2 conditions, the principal components accounted for 36.3% (PC1), 28.2% (PC2), and 16.7% (PC3) of the variance. Interestingly, it was observed that the relationship between the variables differed with the type of storage. In R1 storage, retinol and FRAP showed a positive correlation with a* and C* and a negative correlation with TBARS. In R2 storage, γ-tocopherol and FRAP showed a positive correlation with a* and C* and a negative correlation with TBARS. For both storage conditions, a* and C* parameters showed a negative correlation with TBARS.

## 4. Conclusions

The inclusion of DG in the feeding diets of beef steers increased the content of antioxidant compounds in the diets. This was reflected in a greater content of γ-tocopherol in beef from DG diets, and a numerical increase in retinol content. Although the values of α-tocopherol were below those indicated as optimal for oxidation delay, the values of TBARS observed were far lower than the ones associated with meat rancidity. Therefore, it could be assumed that the antioxidant compounds present in the meat from animals fed with DG exerted a cooperative antioxidant activity.

The meat exposed to refrigerated storage was affected by the conditions of retail display since FRAP and color parameters decreased. The inclusion of DG affected the extent of lipid oxidation. In this regard, the samples from DG-diets showed similar values of TBARS for both storage conditions, and these values were lower than those from 0DG and extended storage. Thus, DG inclusion in finishing diets positively impacts the balance between anti- and pro-oxidant compounds and meat oxidative stability during the display conditions evaluated.

## Figures and Tables

**Table 1:** Composition of the finishing diets on a dry matter (DM) basis (g/100 g DM).

Item	Dietary Treatment ^1^			
	0DG	15DG	30DG	45DG
Ingredient (% DM)				
Cracked corn grain	84	74	64	48
Distiller grains (DG)	0	15	30	45
Soybean meal	10	5	0	0
Alfalfa hay	6	6	6	6
Nucleus ^2^	0.3	0.3	0.3	0.3
Chemical composition (% DM)				
Crude protein	11.72	12.82	14.02	16.80
Fat	4.16	5.37	6.44	7.56
Ash	2.87	3.14	3.27	3.73

^1^ Dietary treatment: 0DG, control, 15DG 15% DG, 30DG 30% DG and 45DG 45% DG. ^2^ Nucleus composition (Vetifarma SA, La Plata, Argentina): vitamin A: 1,000,000 IU/kg; vitamin D3: 200,000 IU/kg; vitamin E: 6500 IU/kg; vitamin B1: 650 ppm; manganese: 12,000 ppm; zinc: 12,000 ppm; copper: 6000 ppm; cobalt: 40 ppm; selenium: 60 ppm; iodine: 200 ppm; with added calcium 0.05%.

**Table 2:** Tocopherol and carotenoid contents (µg/g DM) in feeding diets ^1^.

Item	0DG ^1^	15DG	30DG	45DG	SEM ^2^	*p*-Value	L ^3^	Q
γ-tocopherol	48.37 ^c^	102.43 ^b^	132.80 ^a,b^	160.68 ^a^	9.84	0.0002	<0.0001	NS
α-tocopherol	5.25 ^b^	12.00 ^a^	16.15 ^a^	17.27 ^a^	1.45	0.002	0.0003	NS
β-carotene	1.91 ^c^	1.95 ^b,c^	2.04 ^a,b^	2.11 ^a^	0.02	0.0002	<0.0001	NS
α-carotene	n.d. ^4^	n.d.	n.d.	n.d.				
Lutein	9.62 ^b^	9.53 ^b^	13.18 ^a^	15.01 ^a^	0.75	0.002	0.0004	NS

^1^ Dietary treatment: 0DG, control; 15DG, 15% DG; 30DG, 30% DG and 45DG, 45% DG (%DM basis). ^2^ SEM: standard error of the mean. ^3^ Linear (L) and quadratic (Q) response to DG level. Means in the same row having different letters are significant at the *p* ≤ 0.05 level. NS: no significant. ^4^ n.d., not detectable.

**Table 3:** Content of antioxidant vitamins and oxidation stability in fresh meat samples (LTL) muscle at 72 h post-mortem.

Item	0DG ^1^	15DG	30DG	45DG	SEM ^2^	*p*-Value	L ^3^	Q
Antioxidant compounds (µg/g meat)								
γ-tocopherol	0.83 ^b^	1.02 ^a,b^	1.05 ^a,b^	1.44 ^a^	0.14	0.04	0.007	0.39
α-tocopherol	0.78	0.98	0.98	1.41	0.22	NS	0.06	0.52
Retinol	0.017	0.025	0.030	0.024	0.003	0.099	0.12	0.93
Oxidation stability								
FRAP (eq Fe^+2^/µM)	204	229	226	216	17	NS	0.73	0.33
TBARS (mg MDA/kg meat)	0.39	0.29	0.25	0.22	0.06	NS	0.09	0.27

^1^ Dietary treatment: 0DG, control; 15DG, 15% DG; 30DG, 30% DG and 45DG, 45% DG (%DM basis). ^2^ SEM: standard error of the mean. ^3^ Linear (L) and quadratic (Q) response to DG level. Means in the same row having different letters are significant at the *p* ≤ 0.05 level. NS: no significant.

**Table 4:** Content of antioxidant vitamins and antioxidant stability in LTL muscle at the end of each storage and retail display condition.

Item	Dietary Treatment ^1^					Retail Treatment			*p*-Value		
	0DG	15DG	30DG	45DG	SEM ^2^	R1	R2	SEM	D*R ^3^	D	R
Antioxidant compounds (µg/g meat)											
γ-tocopherol	0.62 ^b^	0.88 ^a^	0.81 ^a,b^	0.75 ^a,b^	0.05	0.78	0.75	0.04	NS	0.04	NS
α-tocopherol	0.52	0.65	0.63	0.51	0.09	0.57	0.58	0.04	NS	NS	NS
Retinol	0.023 ^b^	0.026 ^a,b^	0.030 ^a^	0.024 ^a,b^	0.001	0.027	0.025	0.002	NS	0.03	NS
Antioxidant capacity											
FRAP (eq Fe^+2^/µM)	261	250	261	251	9.20	270 ^a^	241 ^b^	6.82	NS	NS	0.004

^1^ D, Dietary treatment: 0DG, control; 15DG, 15% DG; 30DG, 30% DG and 45DG, 45% DG (%DM basis); R, retail treatment: R1, aerobic exposure for seven days; R2, vacuum-packed conditions for 25 days, plus aerobic exposure for three days. ^2^ SEM: standard error of the mean. ^3^ D*R, dietary treatment x retail treatment interaction. Means in the same row having different letters are significant at the *p* ≤ 0.05 level. NS: no significant.

**Table 5:** Color parameters (CIELab) in LTL muscle at the end of each storage and retail display condition.

Item	Dietary Treatment ^1^					Retail Treatment				*p*-Value		
	0DG	15DG	30DG	45DG	SEM ^2^	R1	R2_a_	R2_b_	SEM	D*R ^3^	D	R
L*	43.24	42.50	41.85	41.37	0.57	40.93 ^b^	42.84 ^a^	42.94 ^a^	0.37	NS	NS	0.0002
a*	17.10	18.32	18.02	18.08	0.47	15.72 ^c^	20.09 ^a^	17.83 ^b^	0.26	NS	NS	<0.0001
C*	19.96	21.45	20.98	21.01	0.45	19.03 ^c^	22.71 ^a^	20.82 ^b^	0.31	NS	NS	<0.0001

^1^ D, Dietary treatment: 0DG, control; 15DG, 15% DG; 30DG, 30% DG and 45DG, 45% DG (%DM basis); R, retail treatment: R1, aerobic exposure; R2_a_, end of vacuum-packed storage (25 days); R2_b_, end of aerobic exposure of R2. ^2^ SEM: standard error of the mean. ^3^ D*R, dietary treatment x retail treatment interaction. Means in the same row having different letters are significant at the *p* ≤ 0.05 level. NS: no significant.

## Data Availability

The data presented in this study are available on request from corresponding author.
